# Homeostasis of the ER redox state subsequent to proteasome inhibition

**DOI:** 10.1038/s41598-021-87944-y

**Published:** 2021-04-21

**Authors:** Yuki Oku, Masahiro Kariya, Takaaki Fujimura, Jun Hoseki, Yasuyoshi Sakai

**Affiliations:** 1 Division of Applied Life Sciences, Graduate School of Agriculture, Kyoto University, Kyoto, 606-8502 Japan; 2 Graduate School of Advanced Integrated Studies in Human Survivability, Kyoto University, Kyoto, 606-8306 Japan; 3 Research Unit for Physiological Chemistry, the Center for the Promotion of Interdisciplinary Education and Research, Kyoto University, Kyoto, 606-8502 Japan; 4 Department of Bioscience and Biotechnology, Faculty of Bioenvironmental Science, Kyoto University of Advanced Science, Kyoto, 621-8555 Japan

**Keywords:** Biochemistry, Cell biology, Molecular biology

## Abstract

Endoplasmic reticulum (ER) maintains within, an oxidative redox state suitable for disulfide bond formation. We monitored the ER redox dynamics subsequent to proteasome inhibition using an ER redox probe ERroGFP S4. Proteasomal inhibition initially led to oxidation of the ER, but gradually the normal redox state was recovered that further led to a reductive state. These events were found to be concomitant with the increase in the both oxidized and reduced glutathione in the microsomal fraction, with a decrease of total intracellular glutathione. The ER reduction was suppressed by pretreatment of a glutathione synthesis inhibitor or by knockdown of ATF4, which induces glutathione-related genes. These results suggested cellular adaptation of ER redox homeostasis: (1) inhibition of proteasome led to accumulation of misfolded proteins and oxidative state in the ER, and (2) the oxidative ER was then reduced by ATF4 activation, followed by influx of glutathione into the ER.

## Introduction

Endoplasmic reticulum (ER) is an organelle responsible for folding and maturation of secretory and membrane proteins, which amount to one third of synthesized proteins. Polypeptides newly synthesized in the ER are folded with the help of molecular chaperones and oxidoreductases such as BiP and protein disulfide isomerase (PDI) family proteins^1,2^. Correctly folded proteins exit from the ER and are transported to the Golgi apparatus. Misfolded proteins are retained and refolded in the ER, and terminally misfolded proteins are retrotranslocated to the cytosol, and degraded by the ubiquitin–proteasome system^3,4^. This process is called ER-associated degradation (ERAD).

ER maintains an oxidative environment suitable for oxidative protein folding^5^. Most of the proteins folded and maturated in ER have intra- and/or intermolecular disulfide bonds that are required for their folding and functions, whereas disulfide bond(s) of terminally misfolded proteins in the ER are reduced by the ER-resident reductase ERdj5 followed by retrotranslocation upon ERAD^6,7^. Therefore, it can be easily assumed that to maintain ER redox homeostasis is essential for protein quality control in the ER.

When stresses exceed the capacity of ER protein quality control system, misfolded proteins accumulate in the ER, a condition referred to as ER stress. ER stress activates adaptive cellular response called unfolded protein response (UPR)^8^, which integrates signal transduction pathways that restore the aberration in ER proteostasis. The UPR is activated by three ER stress sensors, PERK, ATF6 and IRE1^9^, which are successively activated; PERK is first dimerized and phosphorylated, inducing phosphorylation of eIF2α to suppress the general mRNA translation except for the translation of transcriptional factors such as ATF4^10,11^. Next, ATF6 is translocated from the ER to the Golgi and then cleaved by site-1 protease (S1P) and site-2 protease (S2P)^12^. Its cytosolic domain is released from the Golgi membrane and translocated into the nucleus, inducing its target genes encoding ER chaperones^12^. IRE1 is subsequently phosphorylated by oligomerization, activating endoribonuclease functions required to generate the active form of transcriptional factor XBP1s^13^. When these adaptation mechanisms cannot remove the accumulated misfolded proteins sufficiently, cells undergo apoptosis. This has been associated with the pathogeneses of protein-misfolding diseases, including the Alzheimer’s disease and diabetes^14^.

Proteasome, a large protein complex that usually localizes in the cytosol and nucleus, recognizes and degrades unfolded or misfolded proteins tagged with polyubiquitin. Proteasome activity decline by aging and the resulting accumulation of abnormal proteins are known to be associated with the pathogenesis of a variety of diseases including the neurodegenerative diseases^15–17^. Previously, we reported the relationship between dysfunction of proteostasis and intracellular redox state; proteasome inhibition initially damaged mitochondria, resulting in an oxidative state in the cytosol and eventual cell death^18^.

Since ER handles massive proteins, the quality of proteins folded and maturated, and possibly also ER redox state must be strictly controlled^1,2^. However, the mechanism of how the ER redox state is maintained is not understood due to technological difficulties in evaluation of intracellular local redox state through conventional subcellular fractionation and the following biochemical approaches. Previously, we developed a fluorescence redox probe ERroGFP S4 suitable to visualize the redox dynamics of the ER in living cells^19^. Our previous study revealed that overexpression of misfolded proteins in the ER led to oxidation and that treatment of ER stressors led to its reduction^19^. In this study, we followed the dynamics of ER redox state with ERroGFP S4 subsequent to proteasome inhibition, and revealed ATF4-mediated cellular response for maintaining homeostasis of the ER redox state.

## Results

### Time course of ER redox state following proteasome inhibition

**Figure 1:**
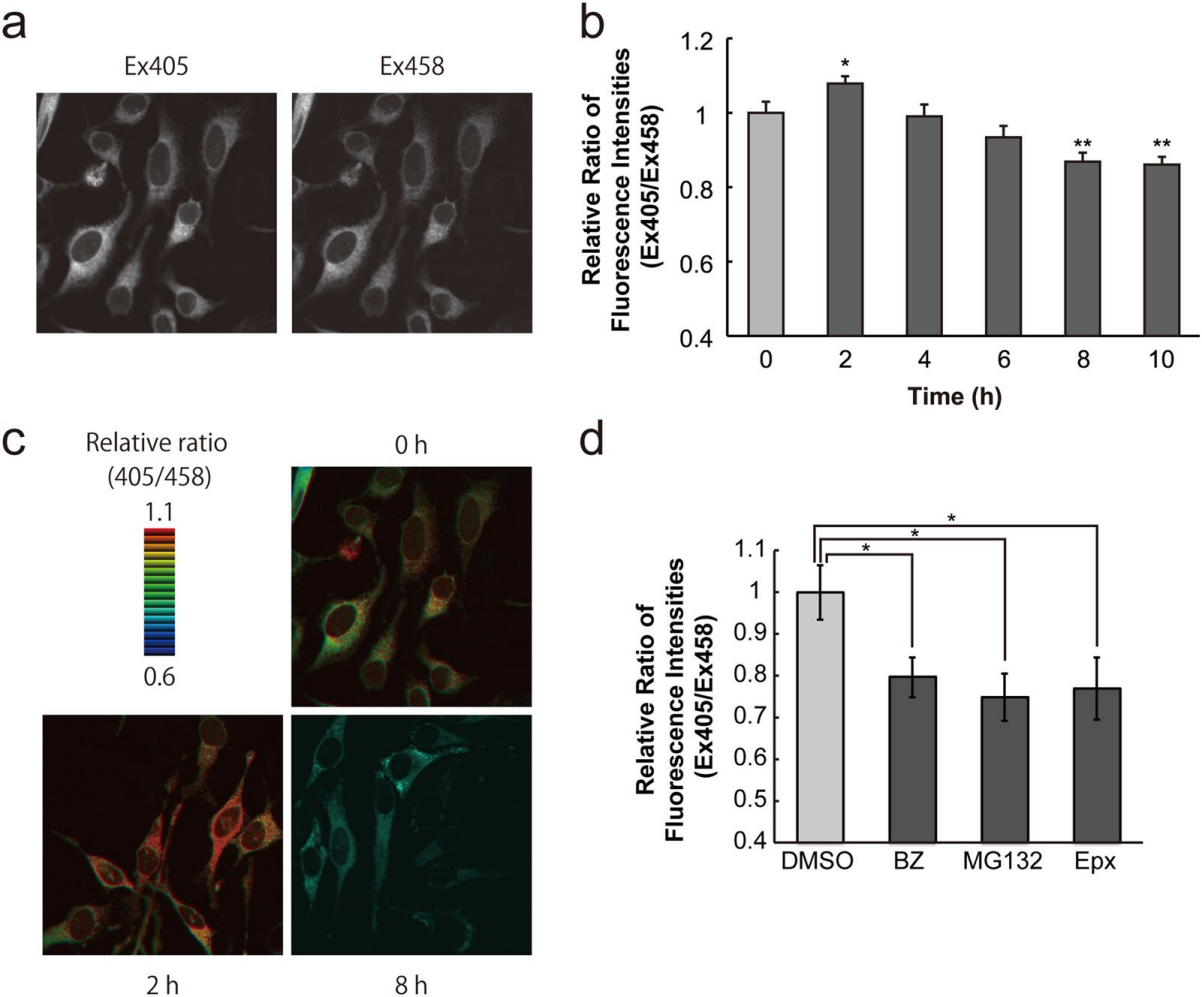
ER redox dynamics following proteasome inhibition. (**a**) Fluorescence images of ERroGFP S4 excited at 405 nm (left) and 458 nm (right) in the stable HeLa cells. (**b**) Time-course of ER redox state after treatment of epoxomicin (Epx) visualized with ERroGFP S4. The relative ratio of fluorescence intensities (Ex405/Ex458) of ERroGFP S4 was quantified at indicated times after treatment with Epx (1 µM). (**c**) Fluorescence ratio (Ex405/Ex458) images of ERroGFP S4 visualizing the ER redox state 0, 2, 8 h after Epx treatment. (**d**) The relative ratio of fluorescence intensities (Ex405/Ex458) from ERroGFP S4 was measured at 8 h after treatment with proteasome inhibitors, bortezomib (BZ) (10 µM) and MG132 (10 µM), and Epx (1 µM). Quantified values are shown as the means ± S.E. of three independent experiments. *P < 0.05 and **P < 0.01 versus control in each condition.

In order to monitor the ER redox state following proteasome inhibition, we used HeLa cells stably expressing ERroGFP S4, which indicates ER redox state in real time^19^. Both of fluorescence images of ERroGFP S4 in the stable cell lines excited at 405 nm and 458 nm showed its ER localization (Fig. 1a and Ref.^19^). The fluorescence intensity ratio (Ex405/Ex458) of ERroGFP S4 increases when ER is oxidized and decreases when reduced^19^. The fluorescence intensity ratio of ERroGFP S4 significantly increased 2 h after treatment with a proteasome-specific inhibitor, epoxomicin (Epx), and after that gradually decreased to the same level as before the treatment at 4 h, and then, decreased further to a significantly lower level (Fig. 1b). The corresponding ER redox dynamics can be visualized by the ratio imaging of ERroGFP S4: yellow to orange in the ER region at 0 h (DMSO treatment), orange to red at 4 h, and green to blue at 8 h (Fig. 1c). These results showed that, after proteasome inhibition, the ER redox state first became slightly oxidized, and then gradually shifted to a reduced state. To confirm that proteasome inhibition leads to ER reduction, ER redox state was measured 8 h after the treatment of three proteasome inhibitors. The fluorescence intensity ratio decreased 8 h after treatment with all of the tested proteasome inhibitors (bortezomib, MG132, and Epx) compared to the control (DMSO treatment), indicating that the ER redox state shifted to a reduced level 8 h after the treatment of proteasome inhibitors (Fig. 1d).

### Mechanism of ER reductive shift following proteasome inhibition

**Figure 2:**
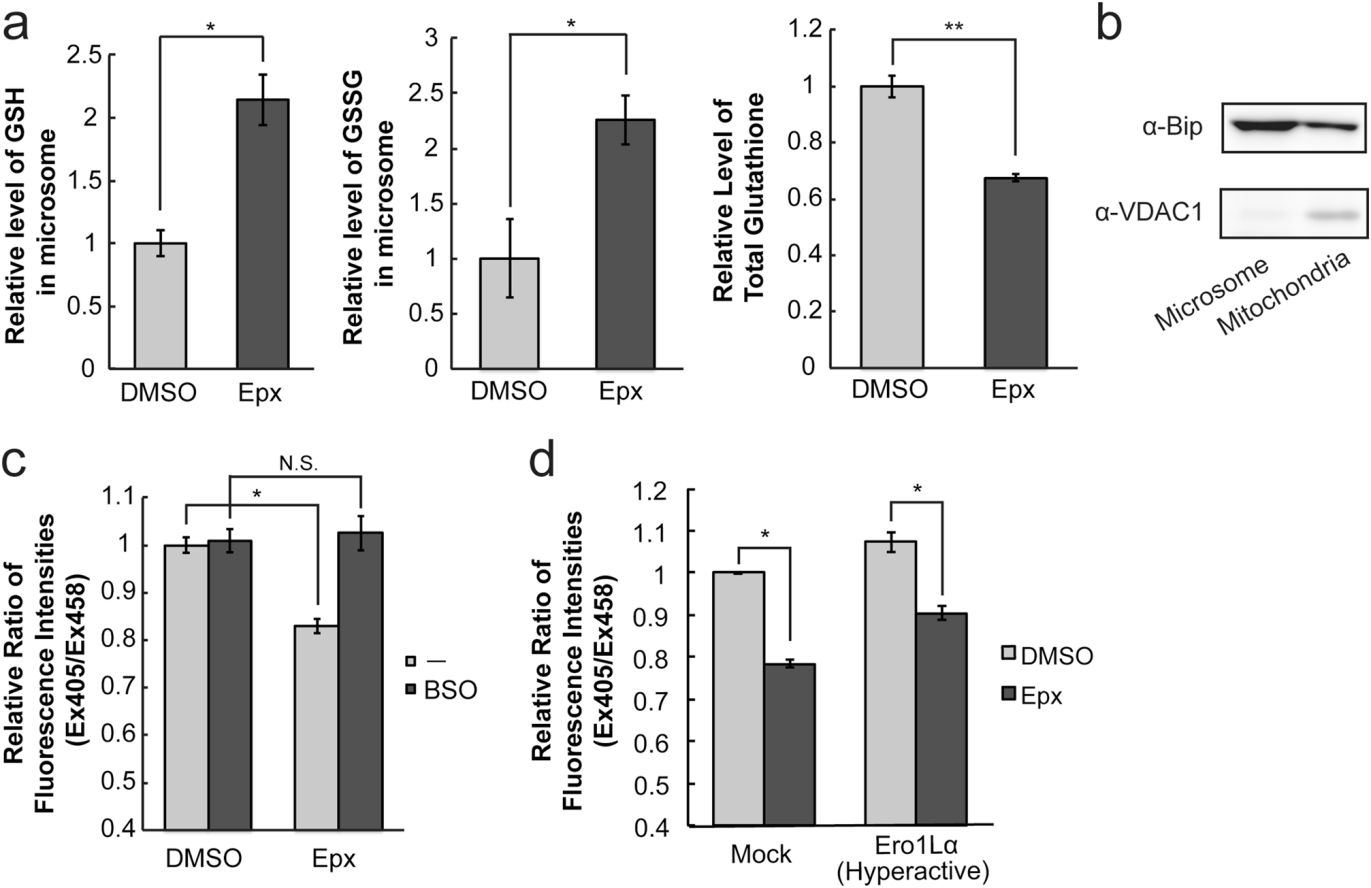
Proteasome inhibition increased the amount of reduced glutathione in the ER. (**a**) The relative level of reduced and oxidized glutathione extracted from microsome fraction and the level of total glutathione extracted from whole cells were determined by LC–MS/MS. HeLa cells were treated with DMSO (control) or Epx for 8 h, and then, the microsome fraction was prepared. (**b**) Western blotting of an ER-localized protein BiP and a mitochondrial outermembrane protein VDAC1 in microsomal and mitochondrial fractions of cells treated with DMSO (control) or Epx for 8 h. (**c**) Effect of pretreatment of a glutathione synthesis inhibitor BSO on ER redox state after proteasome inhibition. HeLa cells stably expressing ERroGFP S4 were pretreated with BSO (100 µM) for 24 h prior to 8 h treatment of Epx. Then, the relative ratio of fluorescence intensities of ERroGFP S4 was determined. (**d**) The effect of overexpression of an Ero1Lα hyperactive mutant (C104A, C131A) on ER redox state after proteasome inhibition. Expression plasmid containing the Ero1 hyperactive mutant gene was transfected in HeLa cells stably expressing ERroGFP S4. The cells at 24 h after transfection were treated with Epx (1 µM) for 8 h, and the relative ratio of fluorescence intensities from ERroGFP S4 was determined. Quantified values are shown as the means ± S.E. of three independent experiments. *P < 0.05 and **P < 0.01 versus control in each condition.

To reveal the molecular basis of the observed ER reduction following proteasome inhibition, we quantified glutathione level in microsomal fraction by LC–MS/MS. Glutathione, the major intracellular redox compound found in mM levels in cells, mainly defines the intracellular redox state by the ratio of reduced (GSH) and oxidized (GSSG) forms, and functions as an intracellular major antioxidant^20^. Both levels of reduced glutathione (GSH) and oxidized glutathione (GSSG) in the microsome fraction increased after proteasome inhibition to more than 2 times higher than that in the control condition (DMSO), although the level of total glutathione in whole cell lysate decreased (Fig. 2a). An immunoblotting analysis of BiP, an ER-localized protein and VDAC1, a mitochondrial membrane protein showed that preparation of microsome fraction was successful, although a portion of BiP was fractionated in mitochondria (Fig. 2b, Supplementary Fig. 1). The result is consistent with reports about localization of BiP in mitochondria^21^. To further investigate involvement of glutathione in the ER reduction after proteasome inhibition, a buthionine sulfoximine (BSO), an inhibitor of glutathione synthesis, was pre-treated prior to Epx treatment. Pre-treatment of BSO (100 µM) for 24 h completely suppressed the reductive shift in the ER induced by proteasome inhibition (Fig. 2c). There was no significant difference in both levels of GSH and GSSG in the microsome fraction between DMSO and Epx treated conditions (Supplementary Fig. 2a). On the other hand, the protein level of rate-limiting enzyme of intracellular glutathione synthesis was unaltered during Epx treatment of 8 h (Supplementary Fig. 2b). In summary, the available quantity of either or both reduced and oxidized glutathione in the cytosol, not increase of glutathione, is required for the ER reduction caused by Epx treatment.

Another possible reason for the ER reductive shift was due to the decrease in oxidizing power in the ER. Ero1L oxidizes PDI, and Ero1L itself is reduced and re-oxidized via its cofactor FAD by O_2_. Thus, turnover of Ero1L produces hydrogen peroxide, and leads to oxidization of the ER redox state^22–24^. The PDI oxidization activity of Ero1Lα is regulated by an ER redox dependent disulfide bond formation via regulatory cysteines (C104 and C131 in hEro1Lα)^25,26^. Monomeric Ox1 (active) and Ox2 (inactive) forms of Ero1α are detected under a non-reducing condition, and the ratio of Ox1 to Ox2 indicates the Ero1Lα activity. Therefore, the redox state of the endogenous Ero1Lα under Epx treatment was analyzed by western blotting following a non-reducing SDS-PAGE. Monomeric Ox1 and Ox2 forms of Ero1α and a disulfide-bonded Ero1Lα complex were detected under non-reducing condition (Supplementary Fig. 3 and Ref.^25,27^). Epx treatment unaltered the activity (ratio of Ox1/Ox2) of the endogenous Ero1Lα, although a covalent Ero1Lα complex decreased. The mutant of hEro1Lα (C104A and C131A) exerts the constitutive activity of PDI oxidation^28^. Overexpression of the constitutive active hEro1Lα mutant showed a slight oxidation of the ER redox state both in control (DMSO treated) and in Epx treated condition, although it did not recover the ER reductive shift following proteasome inhibition at all (Fig. 2d).

In yeast, it was reported that ER stress caused reflux of ER proteins to the cytosol^29,30^. To confirm contribution of reflux of ERroGFP S4 into the cytosol to the ER reduction under proteasome inhibition, subcellular fractionation of cells was performed, resulting that most of ERroGFP S4 were localized in the ER while small portion of ERroGFP S4 were localized in the cytosol fraction (Supplementary Fig. 4). However, the ratio of ERroGFP S4 fractionated into the cytosol under the proteasome inhibition did not increase.

These results suggested that the increase in the amount of glutathione in the ER caused the reductive shift in the ER after proteasome inhibition. While the glutathione level in whole cell decreased, the glutathione level in microsomal fraction increased. Therefore, influx of glutathione from the cytosol into the ER was suggested to increase following proteasome inhibition.

### ATF4-upregulation led to reduction of the ER redox state

**Figure 3:**
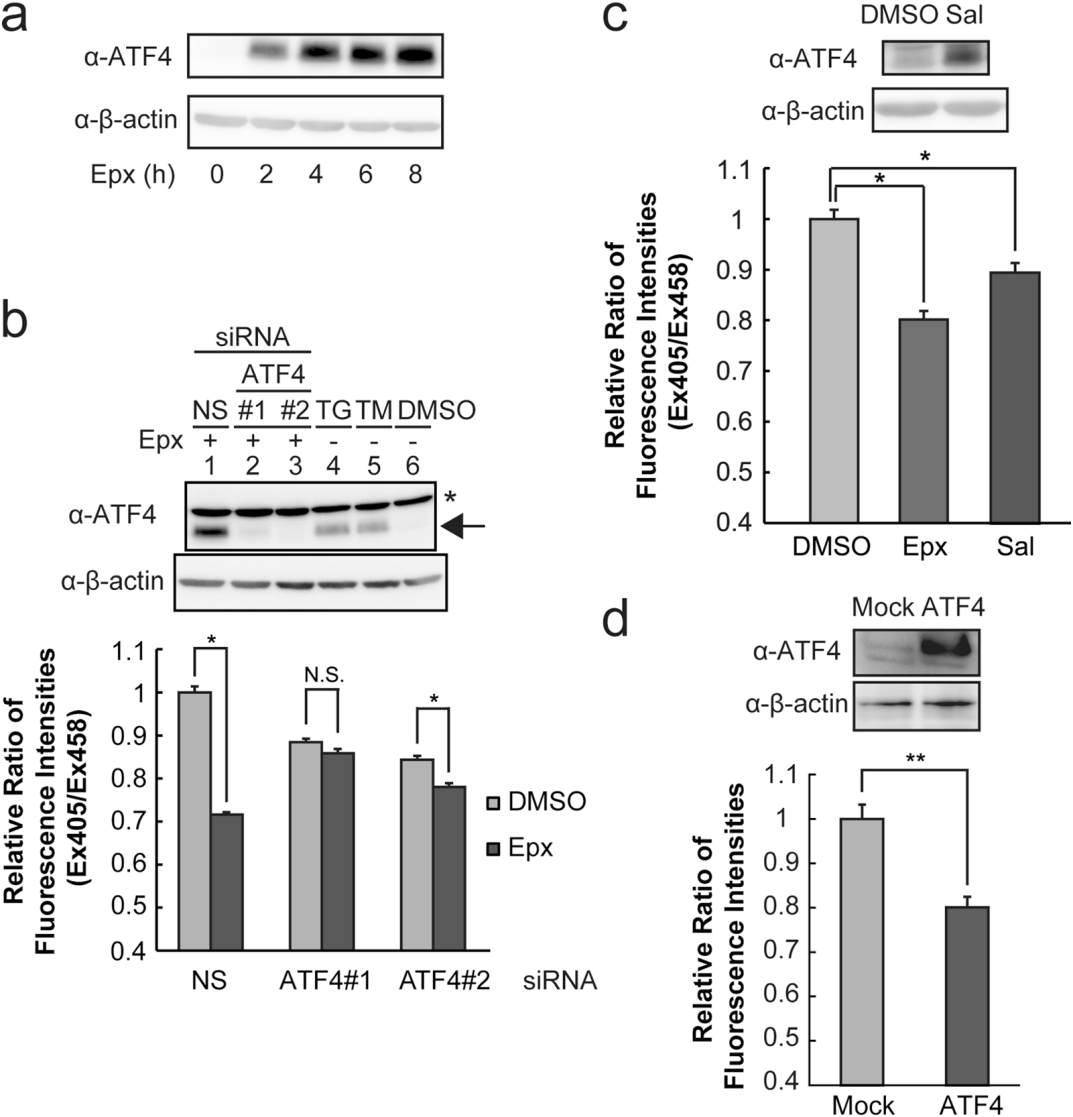
ATF4 was responsible for reduction of the ER redox state after proteasome inhibition. (**a**) Induction of ATF4 proteins after proteasome inhibition was detected at indicated times after Epx treatment. (**b**) Effect of ATF4-knockdown on ER redox state. Western blotting of ATF4 (upper panel) was performed. Thapsigargin (TG) and tunicamycin (TM) were used as positive controls. The relative ratio of fluorescence intensities of ERroGFP S4 (lower panel) was determined. Cells after 48 h of siRNA transfection and 8 h of Epx treatment were lysed or used to determine the fluorescence intensities. (**c**) Effect of ATF4-induction by salubrinal treatment on ER redox state. Salubrinal, an eIF2α dephosphorylation inhibitor (10 μM), was treated for 12 h, which increased ATF4 level (upper panel). After the treatment, the relative ratio of fluorescence intensities was determined (lower panel). (**d**) Effect of overexpression of ATF4 on ER redox state. Expression plasmid containing ATF4 gene was transfected in HeLa cells stably expressing ERroGFP S4. The relative ratio of fluorescence intensities from ERroGFP S4 was determined at 24 h after transfection. The relative ratio was normalized as described in Supplementary Fig. 7. Quantified values are shown as the means ± S.E. of three independent experiments. *P < 0.05 and **P < 0.01 versus control in each condition.

ATF4 induces transcription of the glutathione-related genes including synthesis and transport of glutathione and amino acids^31^. The protein level of ATF4 was induced at 2 h after proteasome inhibition and gradually increased to 8 h (Fig. 3a, Supplementary Fig. 5a). ER stressors, tunicamycin and thapsigargin, also induced ATF4 protein (Fig. 3b, Supplementary Fig. 5b, lane 4–6). XBP1 splicing assay showed Epx treatment weakly induced spliced XBP1, suggesting that ER stress occurred, at 8 h following Epx treatment (Supplementary Fig. 6). To reveal involvement of ATF4 in reduction of the ER redox state after proteasome inhibition, ATF4 was knocked down by transfection of siRNAs specific to ATF4 gene. Proteasome inhibition greatly increased expression of ATF4 upon transfection with the control siRNA, whereas transfection of siRNAs specific to ATF4 gene (siRNA4-1 and 2) hindered the increase in the amount of ATF4 (Fig. 3b upper, Supplementary Fig. 5b, lane 1–3, 6). The siRNAs specific to ATF4 also suppressed the decrease in the relative fluorescence ratio of ERroGFP S4 by Epx treatment, indicating that knockdown of ATF4 abrogated the reductive redox shift of the ER caused by proteasome inhibition (Fig. 3b lower). Knockdown of ATF4 decreased the relative fluorescence ratio of ERroGFP S4 in the control condition, suggesting that ATF4 might be involved in maintenance of the basal ER redox. Transfection of siRNA regardless of specificity to ATF4 increased spliced XBP1 level in Epx treated cells (Supplementary Fig. 6).

Phosphorylation of eIF2α by four kinases including PERK activated by integrated stress facilitates translation of ATF4, whereas it inhibits general translation^32,33^. Salubrinal, an eIF2α dephosphorylation inhibitor, retains phosphorylated eIF2α, resulting promotion of ATF4 translation^34^. Salubrinal treatment increased ATF4 protein expression and decreased the relative fluorescence ratio of ERroGFP S4 after 12 h, although the extent of decrease was less than that after proteasome inhibition (Fig. 3c, Supplementary Fig. 5c). However, salubrinal also was reported to attenuate inflammation by inhibition of NF-kB pathway^35^. Therefore, direct effect of ATF4 on ER redox state was examined by its overexpression. ATF4 overexpression decreased the fluorescence ratio of ERroGFP S4, which indicates ER reduction (Fig. 3d, Supplementary Figs. 5d and 7). Taken together, the increase of ATF4 protein expression level leads to a reductive shift of the ER redox state.

### Effect of reductive shift of ER on protein quality control

**Figure 4:**
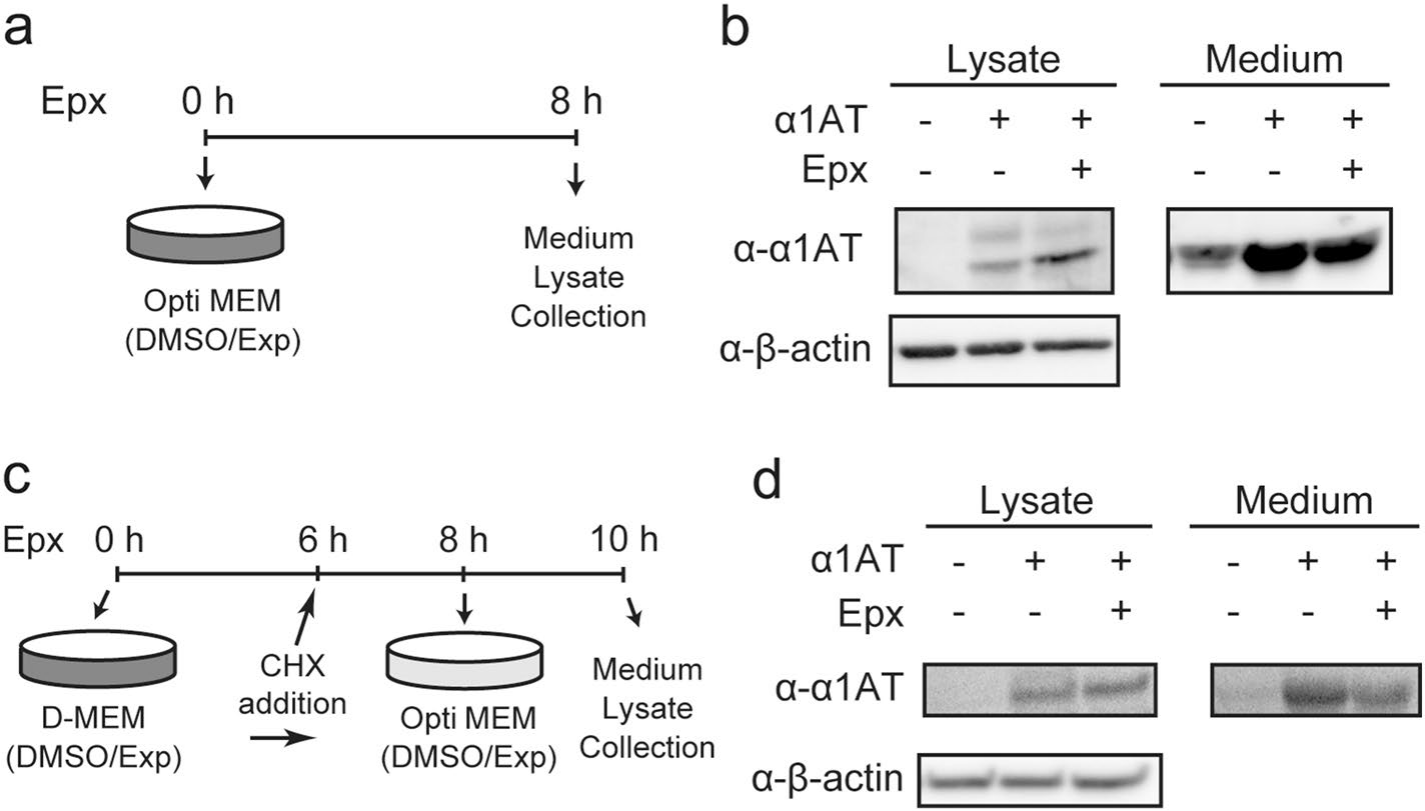
Protein secretion activity was decreased under the reductive redox state in the ER after proteasome inhibition. (**a**, **c**) Experimental design to determine the amount of secreted proteins ((**a**) for (**b**) and (**c**) for (**d**)). Cells were transfected with expression vectors and incubated for 24 h. Then, the medium was exchanged to Opti-MEM containing DMSO and Epx (1 μM), and the cells were incubated for 8 h. In (**c**), after 24 h of the transfection, DMSO or Epx was added and the cells were incubated for 6 h. The medium was then exchanged to a Opti-MEM containing 50 µM cycloheximide (CHX) and, DMSO or Epx, and the cells were further incubated for 2 h. Finally, the medium was exchanged to a new Opti-MEM containing DMSO or Epx. (**b**, **d**) Immunoblotting of secreted or intracellularly retained hα1AT. Secreted proteins were prepared by TCA precipitation and then separated by SDS-PAGE.

ER is in an oxidizing environment suitable for disulfide bond formation of proteins^5^. Therefore, reduction of the ER after proteasome inhibition might affect protein folding and maturation in the ER. To reveal this, the amount of a secreted protein, human α1-anti-trypsin (hα1AT), was investigated after Epx treatment (Fig. 4a). The amount of hα1AT secreted 8 h after treatment by Epx decreased, when compared with the control (DMSO treatment) (Fig. 4b, Supplementary Fig. 8a, medium). On the other hand, there was an increase of intracellular accumulation of hα1AT (Fig. 4b, Supplementary Fig. 8a, lysate). ATF4 protein is induced eIF2α phosphorylation-mediated translational inhibition^32,33^. Therefore, Epx treatment, which induced ATF4 in protein level (Fig. 3a), suggested to cause eIF2α phosphorylation-mediated translational inhibition. In order to escape effect of translational inhibition on the decrease of secreted α1AT under proteasome inhibition, the amount of proteins secreted for 2 h (between 8 and 10 h after Epx treatment when the ER redox state was reductive) after 2 h of cycloheximide treatment, was examined by protocol shown in Fig. 4c. Level of hα1AT secreted in the medium without serum decreased, whereas the amount of hα1AT accumulated in cells increased (Fig. 4d, Supplementary Fig. 8b). Increase of intracellular proteins under proteasome inhibition appears to be due to ERAD inhibition and suppression of secretion.

## Discussion

**Figure 5:**
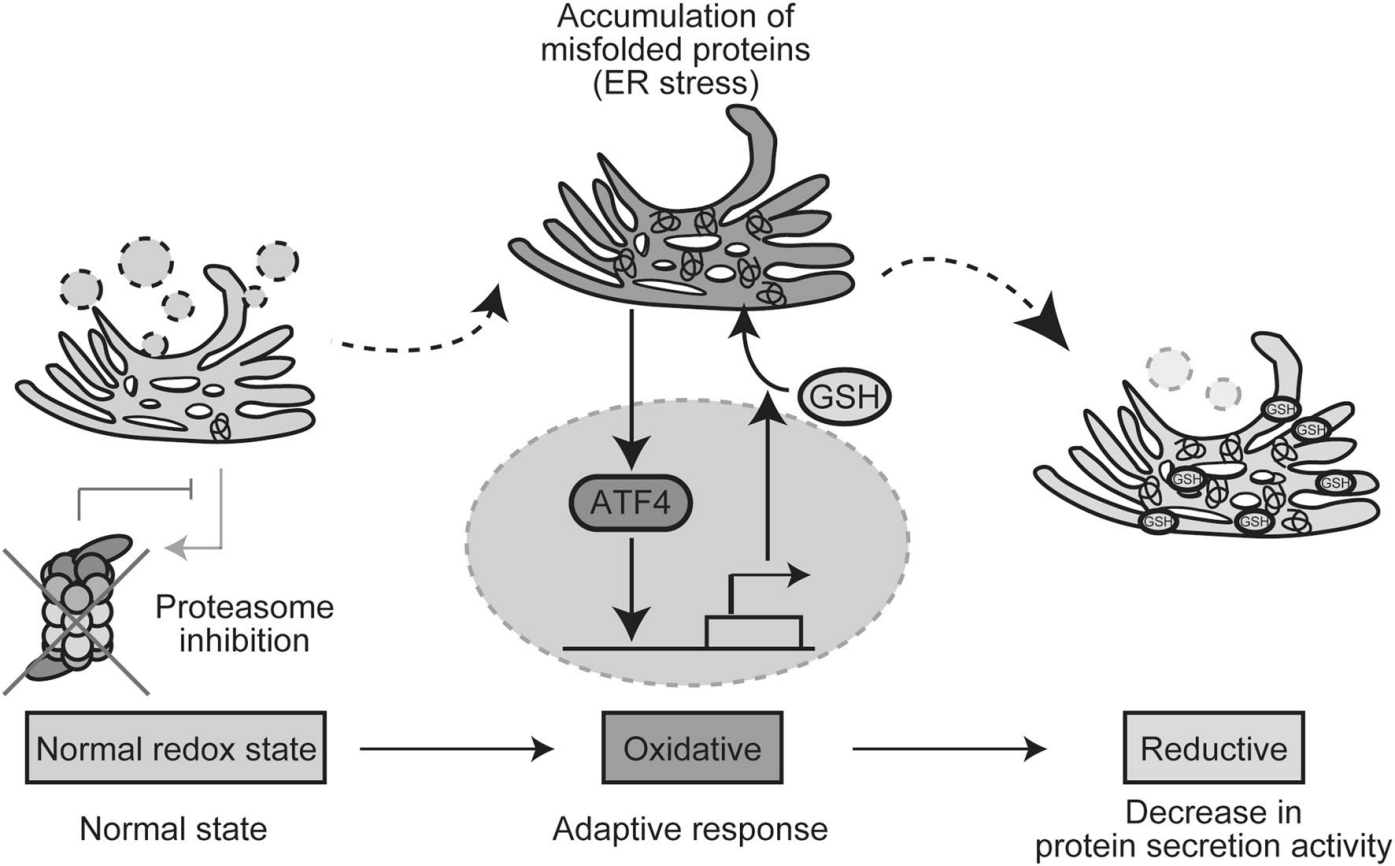
Schematic representation of the redox dynamics in the ER following proteasome inhibition. Proteasome inhibition induces accumulation of misfolded proteins in the ER, namely ER stress. Expression of ATF4 is increased to induce various genes containing glutathione metabolite genes, which causes increase of GSH influx to ER, ER reduction, and inhibition of protein secretion.

The redox state in the ER was reported to change dynamically in response to dysfunction of ER protein quality control^36^. Overexpression of an ER misfolded protein, mIns2 (C96Y) caused an oxidative shift in the ER^19^, whereas treatment by ER stress inducers, tunicamycin or thapsigargin, induced a reductive shift^36^. However, the underling mechanism of this observation has been unclear. In this study, we followed the ER redox dynamics subsequent to proteasome inhibition with the use of an ER redox probe ERroGFP S4^19^. As a result, the ER redox state following proteasome inhibition was initially oxidized up to 2 h, and then, became reduced under control of a transcription factor ATF4 induced by accumulation of the ER misfolded proteins (Figs. 1, 3). ATF4 is known to be necessary for induction of glutathione-related genes. Knockdown of ATF4 or pre-treatment of glutathione synthesis inhibitor suppressed reductive shift of the ER (Figs. 2, 3). Although the total glutathione content in whole cells decreased, the amount of reduced glutathione in microsome fraction increased (Fig. 2a). In addition, the rate-limiting enzyme of glutathione synthesis, GCLC, was not increased in protein level for 8 h of Epx treatment (Supplementary Fig. 2b). Plasma membrane protein xCT (also known as SLC7A11) has been reported to be related to the influx of extracellular cystines into cells under proteasome inhibition in the downstream of ATF4^37,38^. Also, Sec61 has been reported as a transporter of reduced glutathione from the cytosol into the ER in the yeast^39^, although ER glutathione transporter has not been identified in mammalian cells. Upregulation of ATF4 is suggested to induce influx of reduced glutathione or cysteine into the ER through ATF4-upregulated ER transporters, which cause the ER reductive shift (Fig. 5). These might facilitate influx of oxidized and reduced glutathione or cysteine into the ER, followed by reduction by some unknown ER-resident glutathione reductase. We think that ATF4-dependent reduction of the ER redox state is a physiological and adaptive event to maintain its homeostasis against proteasome inhibition.

In *C. elegans*, the ER redox state became reductive upon expression of aggregation-prone poly-glutamate proteins, not only by proteasome inhibition but also during aging^38^. Proteasome inhibition, as an aging model condition, decreased the secreted amount of α1AT (Fig. 4). ER molecular chaperones such as BiP and ER oxidoreductases such as PDI and Ero1L, which support the folding of secreted proteins, are regulated by ER redox states^27,40,41^. Therefore, the reductive shift in the ER caused by proteasome inhibition might affect the activities of these ER resident proteins, leading to impairment of protein folding and secretion.

We still do not know the physiological significance of maintaining the redox state in the ER regarding protein secretion capacity, and whether it is a protective result by cellular response or a negative result by over- or continuous- cellular response remains to be solved. Modulation of the ER redox state may be a potential target against aging and various diseases related with ER stress, although further studies will be necessary for comprehensive understanding of the relationship between the redox state and protein quality control in the ER.

## Methods

### Reagents and antibodies

Epoxomicin, MG132, and bortezomib were purchased from Peptide Institute (Osaka, Japan), Wako Pure Chemical (Osaka, Japan), and Sellect Chemicals (Tokyo, Japan), respectively. Anti-ATF4 (ab184909), anti-VDAC1 antibody (ab154856) (both from Abcam, Tokyo, Japan), anti-β-actin antibody (A5441, Sigma-Aldrich, Tokyo, Japan), anti-insulin (L6B10, #8138) (Cell Signaling Technology, Tokyo, Japan), and anti-BiP (610978, BD Biosciences, Tokyo, Japan) antibodies were used for immunoblotting.

### Cell culture and transfection

HeLa cells were kindly provided by Dr. Kazuhiro Nagata (Kyoto University, Japan). Establishment of HeLa cells stably expressing ERroGFP S4 was described in Ref.^19^. HeLa cells were grown in Dulbecco’s Modified Eagle’s Medium (DMEM) supplemented with 10% (v/v) fetal bovine serum and antibiotics (100 U/ml penicillin and 100 µg/ml streptomycin) under humidified air containing 5% CO_2_ at 37 °C. HeLa cells stably expressing ERroGFP S4 were grown in the same condition as HeLa cells except addition of 1 µg/ml puromycin. Transfection with plasmids was performed using Lipofectamine 2000 (Invitrogen). Transfection with FlexiTube siRNAs specific to human ATF4 gene (QIAGEN, Tokyo, Japan) (siATF4-1: 5′-CAGCGTTGCTGTAACCGACAA-3′, siATF4-2: 5′-AAGCCTAGGTCTCTTAGATGA-3′) or Allstars Negative Control siRNA (control) was performed using Lipofectamine RNAiMax (Invitrogen).

### Construction of plasmids

Human Ero1Lα and ATF4 expression plasmid was constructed as follows: total RNA was extracted from HeLa cells using RNeasy Mini Kit (Qiagen, Tokyo, Japan), treated with DNase I to remove genomic DNA contamination, and reverse-transcribed using ReverTraAce enzyme (TOYOBO, Osaka, Japan). A human Ero1α cDNA with HA-tag and the ER retention signal KDEL sequences at the C-terminus and a human ATF4 cDNA were amplified by PCR from the resulting reverse-transcribed cDNA library and subcloned into pCDNA3.1 (+) (Invitrogen, Tokyo, Japan) with *BamH*I and *Xho*I sites (for Ero1α) or with *EcoR*I and *Not*I sites (for ATF4). The primers used for the amplification were as follows: Ero1α forward: 5′-CGggattcGCCACCATGGGCCGCGGCTGG-3′, Ero1α reverse: 5′-CCGctcgagTTACAGCTCTTGGCGTAGTCGGGCACGTCGTAGGGGTAATGAATATTCTGTAACAAGTTCCTGAA-3′, ATF4 forward: 5′-CCGGAATTCGCCACCATGACCGAAATGAGCTTCCTGAGC-3′, ATF4 reverse: 5′-ATTTGCGGCCGCCTAGGGGACCCTTTTCTTCC-3′. To generate constitutive active Ero1Lα mutant (Ero1-active) gene, the regulatory cysteines (C104 and C131) were substituted into alanines by PCR using PrimeSTAR Max DNA polymerase (Takara-Bio, Shiga, Japan).

### Analysis of the ER redox state with ERroGFP S4

Fluorescence images of HeLa cells stably expressing ERroGFP S4 were acquired by an LSM 510 META confocal microscope (Carl Zeiss, Germany) or an FV3000 confocal laser scanning microscope (Olympus, Tokyo, Japan) and quantified fluorescence intensity ratio (Ex405/Ex458 for the Carl Zeiss lsm) or (Ex405/Ex445 for the Olympus lsm) by using Image J as described previously^19^. Fluorescence intensity ratio images were generated from the acquired fluorescence images using the intensity modulated display mode of MetaMorph imaging software (Universal Imaging Corp., Downingtown, PA).

### Preparation of cell lysates and proteins secreted into medium for Western blotting

For preparation of cell lysates, cells were washed twice with ice-cold phosphate-buffered saline lacking Ca^2+^ and Mg^2+^ (PBS (−)) and then lysed in ice-cold lysis buffer (50 mM Tris–HCl buffer (pH 8.0) containing 150 mM sodium chloride and 1% Triton X-100, freshly supplemented with protease inhibitor cocktail) on ice for 20 min. The cell lysate was centrifuged at 12,000 g for 20 min at 4°C. The supernatant was collected for Western blotting. For preparation of proteins secreted, cell medium was exchanged with Opti-MEM and cells were incubated in Opti-MEM for indicated times. The resultant Opti-MEM was TCA-precipitated. The pellets were washed with acetone twice, and then resolved in PBS (−) for Western blotting.

### Preparation of microsome

Microsome was prepared as described previously^42^. Cells were suspended in buffer I (50 mM Tris–HCl buffer (pH 7.4), 5 mM EDTA), incubated on ice for 10 min, and homogenized by 10 times through a 25-gauge needle. An equal volume of buffer II (buffer I with 880 mM sucrose) was added to the cell homogenate immediately for cancellation of its hypotonic condition. Microsome fraction was prepared by sequential centrifugation (1000 g, 12,000 g, and 120,000 g) of the isotonized cell homogenates.

### Quantification of intracellular glutathione level by LC–MS/MS

Intracellular oxidized and reduced glutathione levels were quantified as previously described^43^. Briefly, a chilled methanol extraction of the whole cells or microsome fractions was freeze-dried and resuspended in 1% acetonitrile, and then applied to a Hydrosphere C18 column (YMC) on a Prominence nano HPLC system (Shimadzu, Kyoto, Japan) in line with a 4000 QTRAP mass spectrometer (AB Sciex Instruments, Foster City, CA) equipped with Hydrosphere C18 column (YMC).

### Statistical analysis

All the data processing and statistical analyses were conducted with Microsoft Excel (Microsoft, USA) or statistical software R. Mean values were considered as representative and all the values were calculated based on independent 3 trials. Welch’s t-test or Wilcoxon rank sum test was applied when comparing the average values of two different groups and Steel’s test was applied when a multiple-comparison was performed for a control condition. *p* < 0.05 threshold was considered to express the significant difference.

## Supplementary Information

Supplementary Figures.

## Data Availability

All data in this study are available in the main text.
