# Supplementary material for: Homeostasis of the ER redox state subsequent to proteasome inhibition
Source: Sci Rep. 2021 Apr 21;11:8655. doi: 10.1038/s41598-021-87944-y (PMC8060268; doi:10.1038/s41598-021-87944-y)
Supplement: Supplementary file 1 — Supplementary Figures. [file 41598_2021_87944_MOESM1_ESM.pdf]

Supplementary Information (Supplementary Figure 1-8)

Homeostasis of the ER Redox State subsequent to proteasome inhibition

Yuki Oku[1,2], Masahiro Kariya[1], Takaaki Fujimura[1], Jun Hoseki[1,3,4] & Yasuyoshi Sakai[1,2,3]

[1]Division of Applied Life Sciences, Graduate School of Agriculture, Kyoto University, Kyoto 606-8502, Japan, [2]Graduate School of Advanced Integrated Studies in Human Survivability, Kyoto University, Kyoto 606-8306, Japan, [3]Research Unit for Physiological Chemistry, the Center for the Promotion of Interdisciplinary Education and Research, Kyoto University, Kyoto 606-8502, Japan. [4]Department of Bioscience and Biotechnology, Faculty of Bioenvironmental Science, Kyoto University of Advanced Science, Kyoto 621-8555, Japan.

Correspondence and requests for materials should be addressed to J.H. (email: hoseki.jun@kuas.ac.jp)

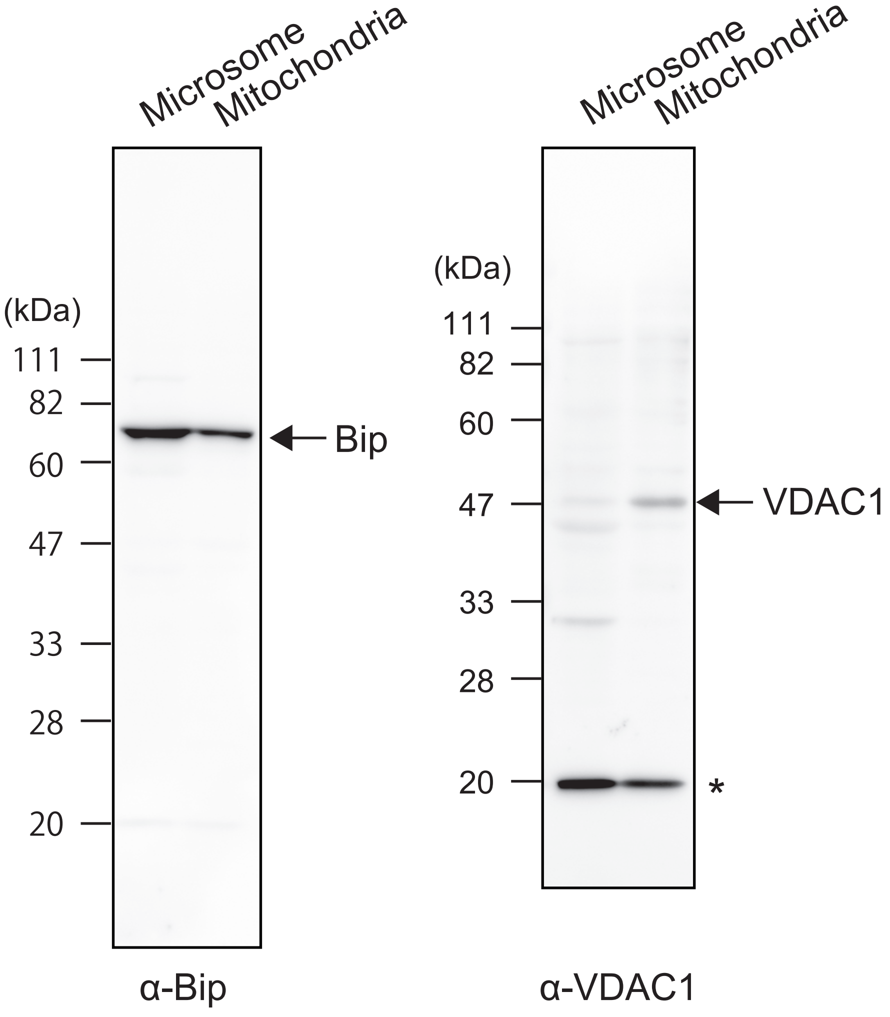

Supplementray Figure 1. **Full blots of Fig. 2b**.

*: Non-target protein

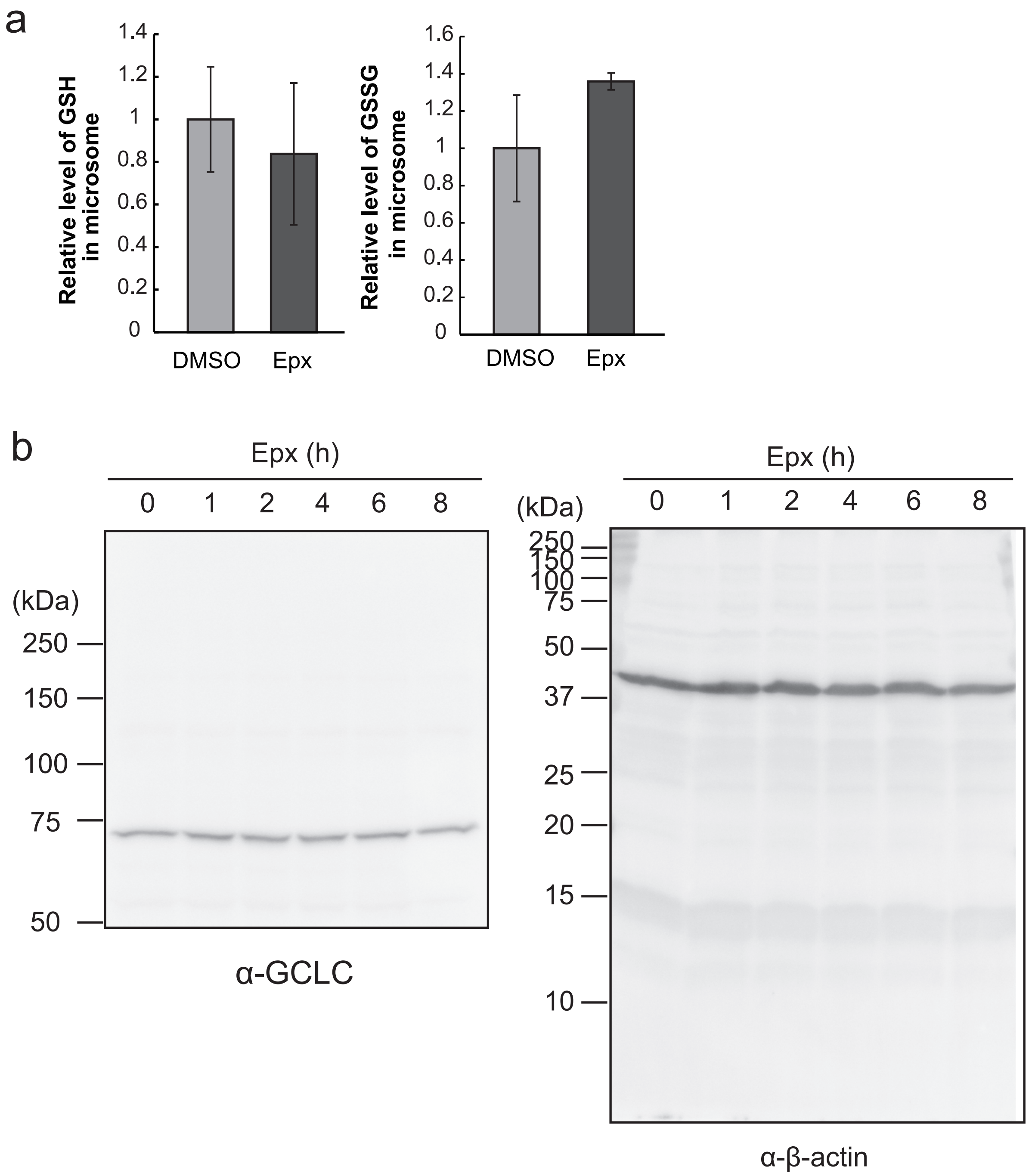

Supplementray Figure 2. **Relationship between ER reduction caused by Epx treatment and glutathione.** (a) **The relative level of reduced and oxidized glutathione in cells pretreated with BSO.** The relative level of reduced and oxidized glutathione extracted from microsome fraction and the level of total glutathione extracted from whole cells were determined by LC-MS/MS. HeLa cells were pre-treated with BSO for 24 h and then treated with DMSO (control) or Epx for 8 h. After that, the microsome fraction was prepared. (b) **Western blotting of GCLC under Epx treatment.**

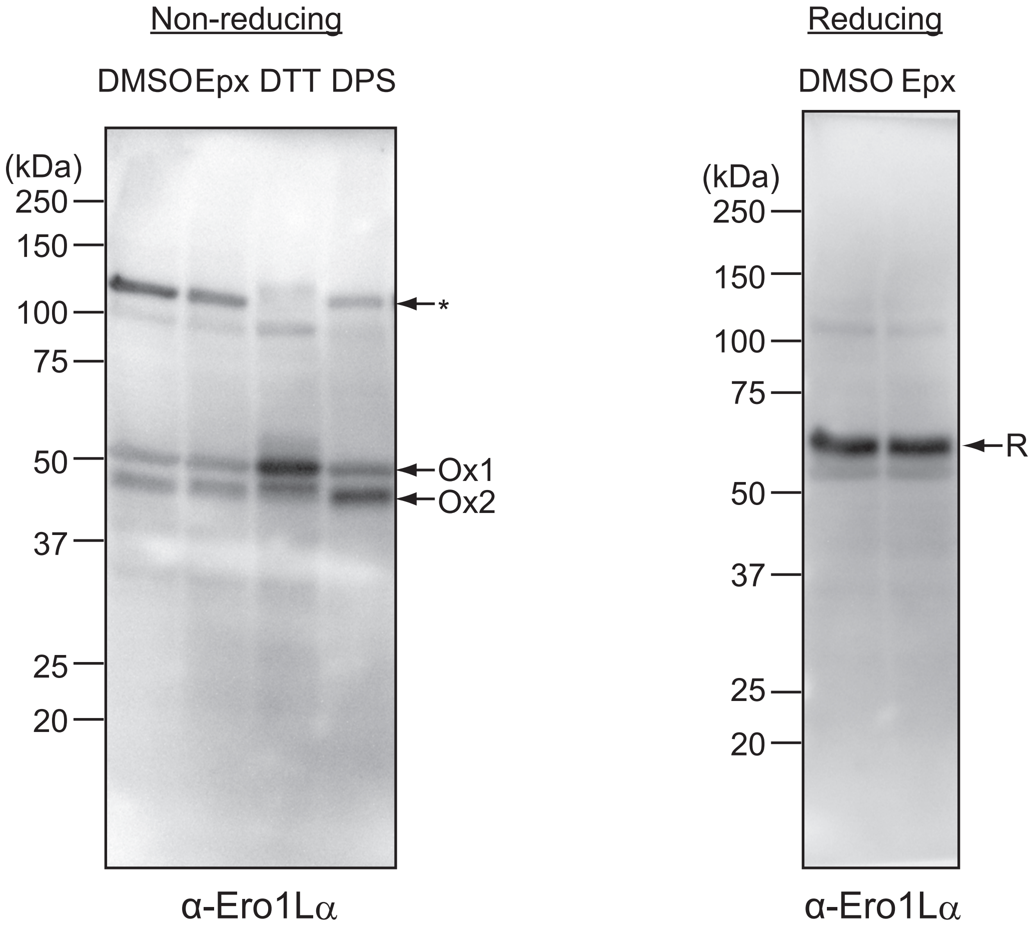

Supplementray Figure 3. **The redox state of the endogenous Ero1Lα under proteasome inhibition.** HeLa cells treated with DMSO or Epx for 8 h, or 0.5 mM DPS (as a control of an oxidized condition) or 5 mM DTT (as a control of a reduced condition) for 5 min were TCA precipitated. The precitated proteins were solubilized in a buffer containing NEM and analysed by western blotting using anti-Ero1Lα (#3264) (from Cell Signaling Technology, Tokyo, Japan) following a non-reducing (left) or a reducing (right) SDS–PAGE. Ox1: active form, Ox2: inactive form, R: reduced form, *: a covalent Ero1Lα complex.

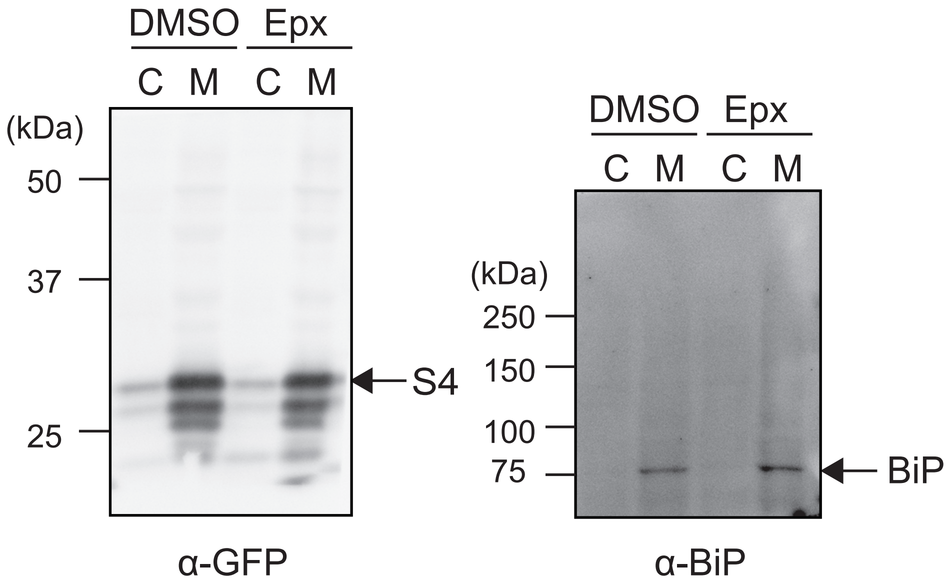

Supplementray Figure 4. Western blotting of ERroGFP S4 and an ER-localized protein BiP in cytosolic (c) and microsomal (m) fractions of cells treated with DMSO (control) or Epx for 8 h.

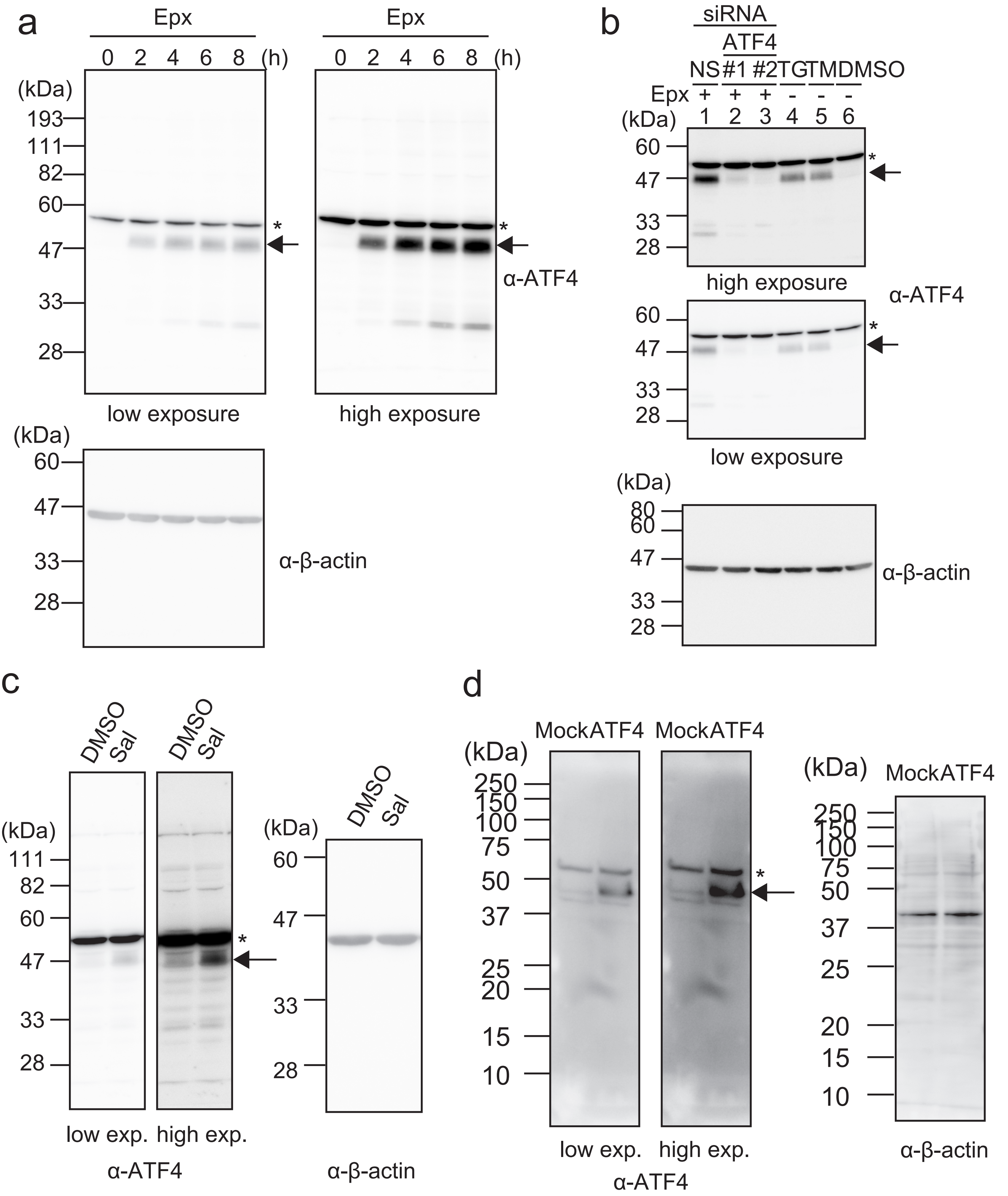

Supplementray Figure 5. (a) Full blots of Fig. 3a, (b) Full blots of Fig.3b. (c) Full blots of Fig. 3c. (d) Full blots of Fig. 3d. *: non-target protein

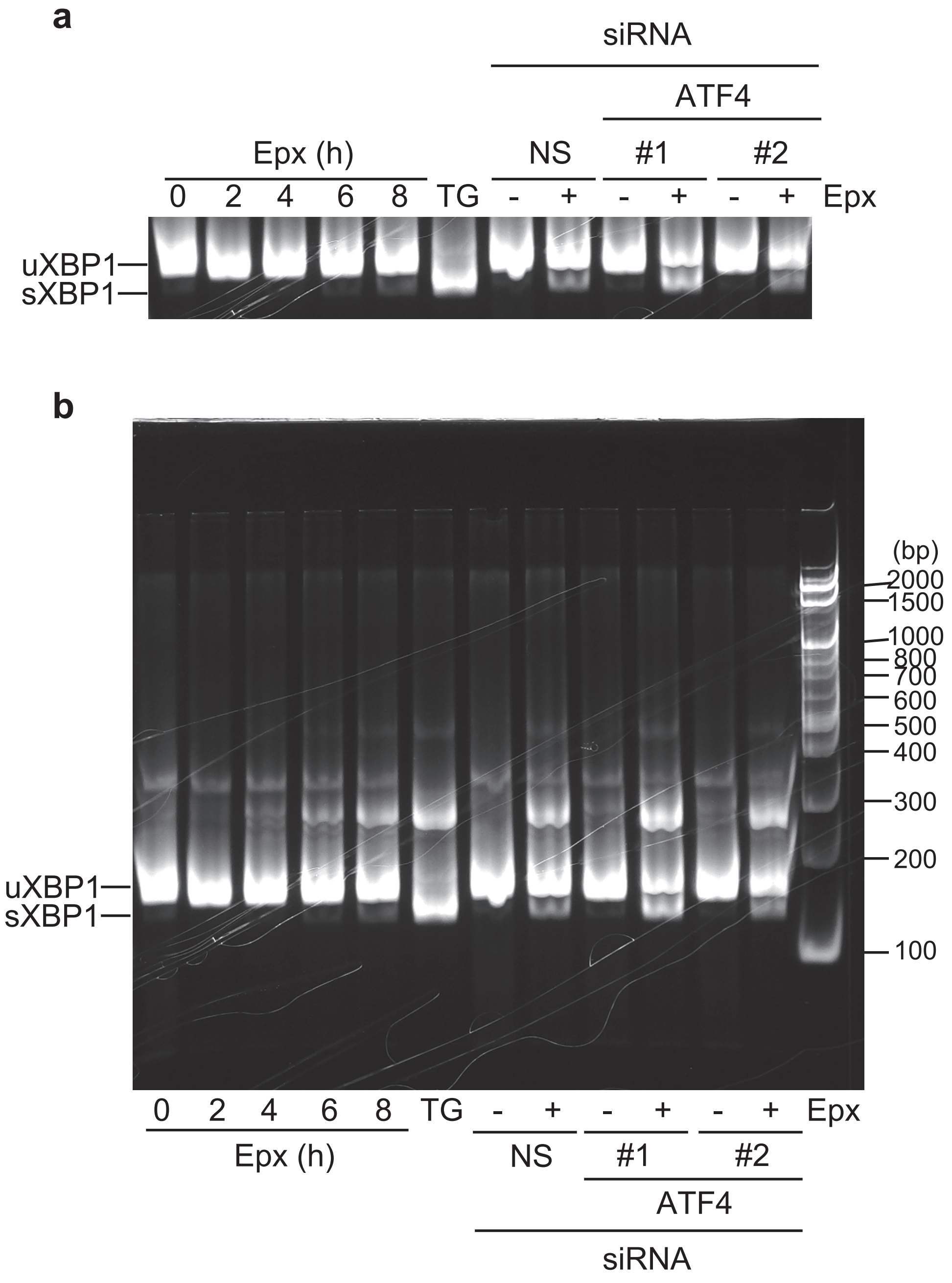

Supplementray Figure 6 **XBP1 splicing assay under Epx treatment.** Cropped gel (a) and full gel (b). Cells were treated with Epx for indicated times or treated for 8 h following 48 h of siRNA transfection. Thapsigargin (TG) with 8 h treatment was used as a positive control. Total RNA was extracted from the cells and then amplified with a pair of primers corresponding to the nucleotides (forward:5’ -GGAGTTAAGACAG-CGCTTGGGGA-3’ , forward) and (reverse: 5’ -TGTTCTGGAGGGGTGACAACTGG-G-3’) of human XBP1 unspliced cDNA. PCR products were analyzed by 5% acrylamide gel electrophoresis. uXBP1: unspriced XBP1, sXBP1: spliced XBP1.

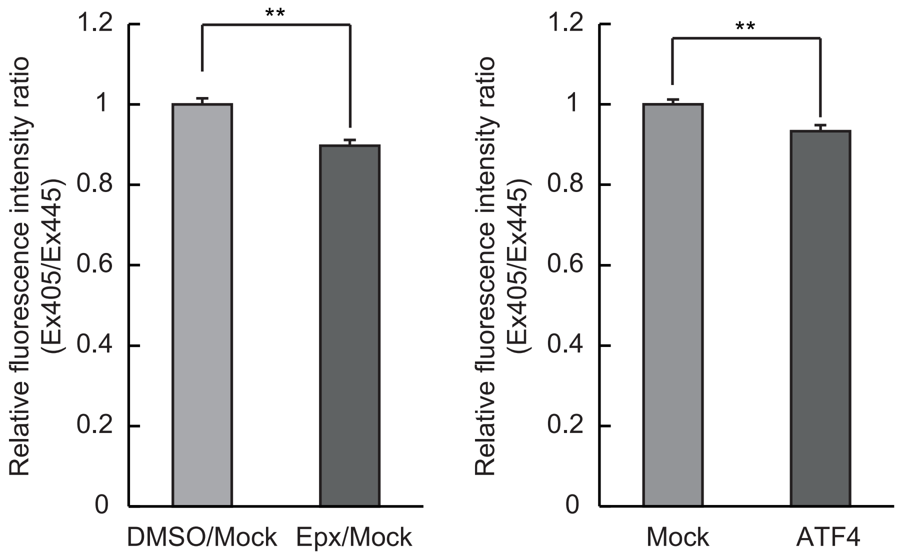

Supplementray Figure 7. **Effect of overexpression of ATF4 on ER redox state.** An expression plasmid with or without ATF4 gene was transfected in HeLa cells stably expressing ERroGFP. The relative ratio of fluorescence intensities from ERroGFP S4 was determined at 24 h after transfection using an Olympus' s lsm (Ex405/Ex445) (right panel). For keeping consistency of data in this paper, the fluorescence ratios measured by the Olympus' s lsm were normalized to those measured by a Carl Zeiss' s lsm in Fig. 1d, HeLa cells stably expressing ERroGFP, which were transfected with the expression plasmid without an insert gene, were treated with DMSO or Epx for 8 h and then the relative ratio of fluorescence intensities (Ex405/Ex445) from ERroGFP S4 (left panel). The normalized data were shown in Fig. 3d. Quantified values are shown as the means ± S.E. of three independent experiments. $^{**}P < 0.01$ vs. control in each condition.

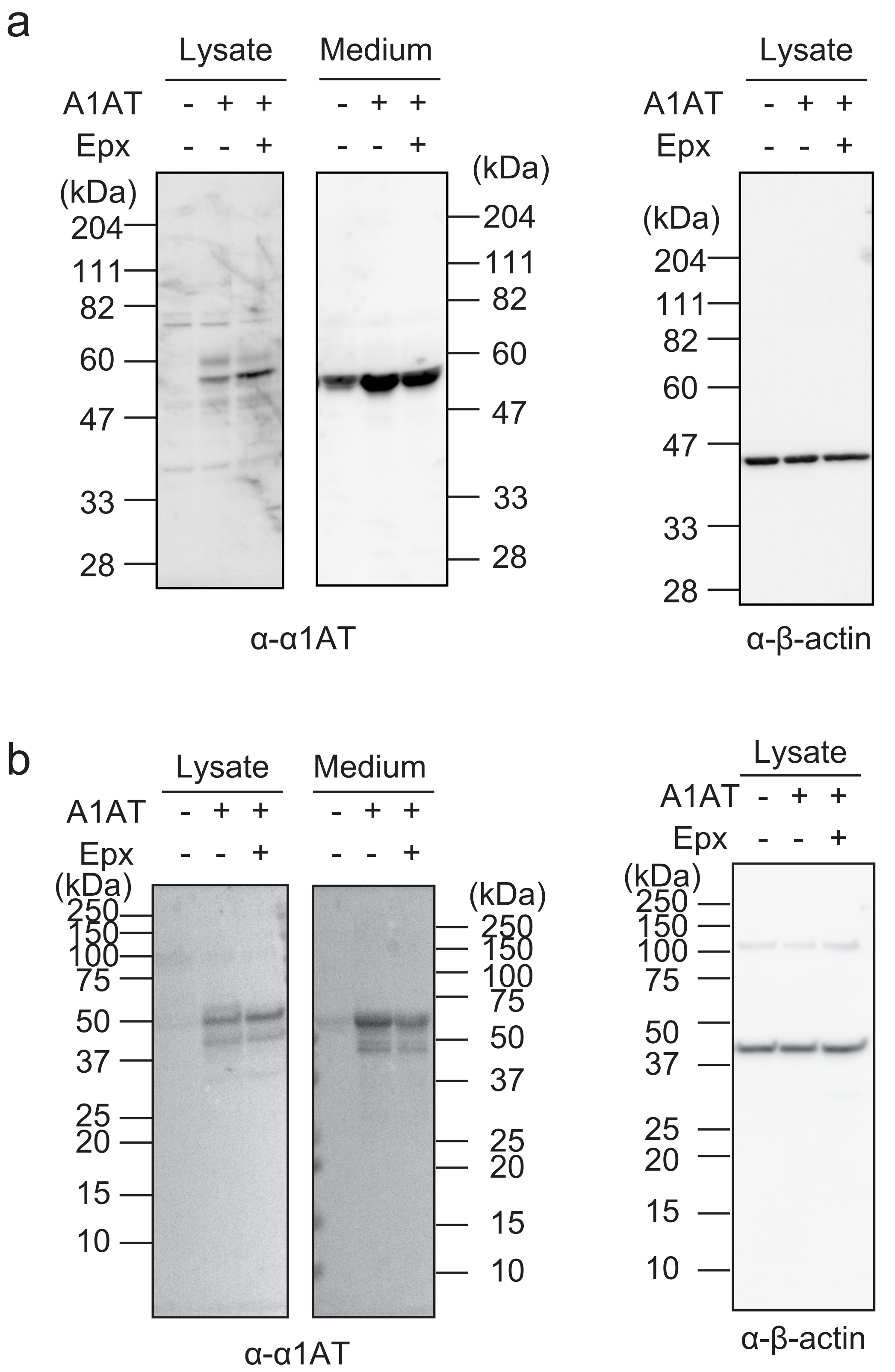

Supplementray Figure 8. (a) Full blots of Fig. 4b, (b) Full blots of Fig. 4d.
